# Common mental disorders through the eyes of German employees: attributed relevance of work-related causes and prevention measures assessed by a standardised survey

**DOI:** 10.1007/s00420-019-01414-7

**Published:** 2019-02-07

**Authors:** Stephanie Burgess, Florian Junne, Eva Rothermund, Stephan Zipfel, Harald Gündel, Monika A. Rieger, Martina Michaelis

**Affiliations:** 1 Institute of Occupational and Social Medicine and Health Services Research, University Hospital Tübingen, Wilhelmstraße 27, 72074 Tübingen, Germany; 2 University Hospital for Psychosomatic Medicine and Psychotherapy, Ulm, Germany; 3 Department of Psychosomatic Medicine and Psychotherapy, University Hospital Tübingen, Tübingen, Germany; 4 Leadership Personality Centre Ulm (LPCU), University of Ulm, Ulm, Germany; 5 Research Centre for Occupational and Social Medicine (FFAS), Freiburg, Germany

**Keywords:** Common mental disorders, Employee perceptions, Work-related risk factors, Prevention measures, Cross-sectional survey

## Abstract

**Purpose:**

Common mental disorders (CMDs) are becoming increasingly relevant in the working world. Numbers of risk factors have been confirmed by mostly correlative cross-sectional studies. Comprehensive and effective prevention is urgently needed. There is little knowledge about employees’ own perceptions on causes of CMDs and prevention measures. Therefore, a survey was conducted in 2016.

**Methods:**

A standardised instrument was developed for an online survey in a commercial access panel, targeted employees in different job types. We assessed two outcomes: perceived relevance of (1) work-related demands to the development of CMDs, and (2) prevention approaches in the workplace and on individual and societal levels. Possible predictive aspects were analysed exploratively by multivariate linear regression analysis.

**Results:**

The response rate was 75% (*n* = 610). Job types were categorised as “blue”, “grey” and “white-collar” workers (*n* = 193, 169, 248). The majority of respondents rated both outcomes consistently as “quite” or “very relevant”; societal prevention strategies were more relevant for non-white-collar workers. Perceived relevance of individual predisposition to develop a CMD was the strongest predictor for both outcomes, indicating the perception that people with higher personal vulnerability might suffer a higher strain from work-related risk factors than others.

**Conclusion:**

We assume that participants in our survey judged the relevance of work-related causes of CMDs independently of their own workload. The perceived relevance of prevention measures in different areas is consistent with official guidelines. A possible selection bias due to characteristics of access panel collectives and own direct or indirect experiences with CMD should be critically questioned.

**Electronic supplementary material:**

The online version of this article (10.1007/s00420-019-01414-7) contains supplementary material, which is available to authorized users.

## Background

An increasing number of scientific studies have shown a significant correlation between common mental disorders (CMDs) and working conditions such as poor work organisation, inadequate leadership from supervisors, other difficult social relations in the work environment, or a gap between workplace demands and employees’ competencies (Rothe et al. 2017; Harvey et al. 2017; Theorell et al. 2015; Fernandes and Pereira 2016; Nieuwenhuijsen et al. 2010; Finney et al. 2013; Nielsen et al. 2014; Gregersen et al. 2011; Warszewska-Makuch et al. 2015).

Moreover, physically active workers, so-called “blue” and “grey-collar workers” in the industrial and craft sectors, have to cope with different psychological distress compared to “white-collar workers” employed predominantly in offices. The activities of the former are often characterised by monotony and lack of scope for action. White-collar workers, on the other hand, are often exposed to deadlines, performance pressure and multitasking. However, distress about social relationships in the workplace can be observed across all sectors (Lohmann-Haislah 2012).

In parallel, private risk factors for CMDs, such as lack of social support or family cohesion, have been discussed (Repetti et al. 2002), as has the individual predisposition to mental disorders, for which a large number of gene variations and thus also a high heredity have been claimed; for example, for major depression, the epigenetic part is discussed as being about 40%, for unipolar disorders it is approximately 15% (Januar et al. 2015; Gatt et al. 2015; Sonnenmoser 2004). Another point might be the interaction between personal vulnerability and strain caused by life experiences. According to the diathesis–stress model, some people are more susceptible to disorders than others (Belsky and Pluess 2009).

The findings outlined above are often used to explain the rising numbers of incapacity for work and/or disability pensions due to CMDs—namely depression, anxiety, somatoform and adjustment disorders—recorded by the statutory health and pension insurance providers in Germany (Hofmann 2012). Sick days have increased steadily between 2005 and 2015, and similarly the frequency of treatment has also increased by 40% in secondary data analysis of German health insurance data (Busch 2012, 2016). Meanwhile, the average duration of sick leave due to CMDs is almost three times higher than for physical ailments based on analyses of data from five of the biggest German statutory health insurances, e.g. TK, AOK (Unger and Richter 2015).

On the other hand, there is insufficient empirical evidence of a real increase in CMDs among the general population; prevalence rates from epidemiologic studies using surveys have remained approximately the same since the 1990s (Richter and Berger 2013). Causes of the phenomenon are attributed to higher awareness of symptoms among professionals such as family doctors or the decreasingly taboo nature of a CMD diagnosis (BPtK 2010). CMDs followed by incapacity for work or disability have financial implications for companies and the social security system, not to mention the harm they cause to the individuals concerned and the risk of developing a long-term condition (Rothermund et al. 2014). In addition, the numbers of incapacity for work and/or disability pensions may depend on the social security benefits provided. Studies show that the return-to-work is also dependent on the extent of compensation paid by the social security system, as well as on the bureaucratic effort required to receive compensation (Anema et al. 2009; Collie et al. 2016).

Despite these scientific findings, data from the people affected themselves are still lacking. To our knowledge, there are hardly any studies on the opinions of employees about common mental disorders at work.

In the prevention of CMDs, the workplace is increasingly recognised as an important setting for health promotion, not only for addressing work-related health problems, but also personal problems that may become visible or be exacerbated within the working environment. Measures of primary prevention (e.g. change in working conditions or behavioural prevention measures to reduce occupational stress (Tan et al. 2014)), secondary prevention (early detection of CMDs and early intervention to avoid long-term or chronic progression) and tertiary prevention (return-to-work after long-term sick leave) seem to be effective under certain circumstances (van Beurden et al. 2015). Overall, prevention activities in the workplace have for a long time been focussed on physical rather than on mental health and to date, high-quality intervention studies addressing mental health are limited and the transfer from research into practice is often insufficient (Reavley et al. 2014).

Although there are numerous scientific investigations using tools to assess psychological work demands and strain (Richter 2010) or the effectiveness of prevention strategies, little information is available about the target groups’ own perceptions of their relevance to developing or preventing CMDs. Against this backdrop, we conducted a standardised survey in October 2016, following our PHOEBE I-study in 2014. In that study, we had investigated health care providers and human resource managers with partly comparable questions (Michaelis et al. 2016; Rothermund et al. 2018). The main research questions in this part of the study were:

What do employees think of the relevance of:

1. different work-related demands and individual predisposition to the development of CMDs among employees, and
2. various prevention activities at company, individual and societal levels for decreasing the risk of CMDs and their consequences?

Moreover, the answering patterns of respondents with different job types and other personal characteristics were of particular interest. Besides content-related questions, we were interested in the psychometric properties of the self-constructed items and their structural validity.

## Methods

The investigation had a target size of about 600 respondents and was conducted as an online survey in a commercial access panel (https://www.researchnow.com). This had the advantage of systematic access to volunteers with a broad range of professions. Participants were rewarded with small non-monetary incentives (shopping vouchers). Inclusion criteria were an age between 18 and 65 years and being employed in any economic sector with the exception of agriculture/fishing and mining/quarrying due to the different operational contexts of employment in the latter and the predominantly self-employed status of farmers.

Job types were classified into three categories, distinguishing service occupations (grey-collar workers (Silaski 2012)) from office-based and industrial/craft occupations (white- and blue-collar workers (Stevenson 2010)) as an indicator of the different demands in various occupational fields. Sectors were defined as follows:

1. Blue-collar workers: Manufacturing/processing/craft occupations.
2. Grey-collar workers: Care, support and medical assistance occupations, service occupations in the areas of facility management (caretakers, building cleaning and cleaning activities, security services), warehouse/logistics/transport, catering/hotel industry, trade.
3. White-collar workers: Office, social and educational professions.

Our recruitment sample was first stratified according to the three job types, age and gender. Non-target participants, refusers and study dropouts were replaced by randomly selected new participants in the respective job-type groups.

All in all 1104 participants were contacted. Incomplete data sets could be avoided through completion control implemented in the online tool. “Speeder” (i.e. people answering too fast) or participants with conspicuous response behaviour were excluded by the data provider. A dropout analysis was undertaken, controlling for job type and systematic termination at potentially critical items.

### Questionnaire operationalisation

For respondents, a definition of CMDs was given in the questionnaire as following: “By common mental disorders we mean all complaints and diseases connected with the psyche, e.g. depression, burnout, compulsive behaviour, anxiety, eating or addiction disorders. Please also consider “psychosomatic” complaints (those which also affect the body, although no physical cause can be found, e.g. chronic exhaustion or recurring sleep disorders). Not only severe impairments, but anything impairing everyday life or work so it cannot be continued as before”.

The instrument was operationalised with a selection of self-constructed items partly based on our PHOEBE I-study among healthcare providers and human resource managers mentioned above (Michaelis et al. 2016). Its usability was pretested with 11 employees. The first outcome “Perceived relevance of work-related CMD causes” included 15 items addressing work-related demands:

- four items addressing work content: quantitative and qualitative job demands, emotional demands in the workplace, and influence and development potential on the job; these were directly derived from the titles of the respective scale dimensions in the German Copenhagen Psychosocial Questionnaire (http://www.copsoq.de; Nübling et al. 2006);
- three items addressing work organisation, derived from several sources (Nübling et al. 2006, 2010; BAuA 2014): organisation of work processes, working time organisation, and work-privacy conflict;
- seven items addressing interpersonal relations and leadership, mainly following the names of COPSOQ dimensions or items (Nübling et al. 2006; Pejtersen et al. 2010): communication culture in the team/in the company, social relationships in the workplace, leadership quality of superiors, leadership culture in the company, injustice, lack of appreciation at work;
- a global item assessing the physical work environment (Nübling et al. 2015).

The second outcome “Perceived relevance of prevention strategies for avoiding CMDs” was operationalised with 17 items covering workplace prevention analogous to the PHOEBE I-study, and a further 11 items covering individual prevention as well as 5 items related to societal prevention. Additionally, in a 5-point Likert-scaled global item, we asked where prevention should start primarily—in the workplace or in private life (response options: exclusively/mostly/both equally). For more details and all items of both outcomes, see Online Resource 1, Table O1–O4.

Items to assess sample characteristics were derived from various sources (* indicates self-constructed items):

- *Sociodemographic variables* Age, gender, origin*, education (RKI 2012).
- *Occupational characteristics* Occupational status as employee, job tenure, work setting experience (number of employers worked for) and company size of current employer, teamwork in the workplace (Evaluation of Social Systems, EVOS Scale (Aguilar-Raab et al. (2015)).
- *Job satisfaction* Global item from the German COPSOQ (Nübling et al. 2006).
- *Work ability* Global items “Mental work ability”, “Physical work ability” (dimension 2), and “Work ability in 2 years from now” (dimension 6) of the Work Ability Index (WAI (Tuomi et al. 2001)).
- Experiences with CMDs* Two global items related to personal experience with CMDs and experience within one’s own social environment.
- Attitudes related to CMDs and prevention* Six global items considering the general open-mindedness of respondents, and their social and vocational environment, namely the perceived willingness of employers in general/of the participant’s employer to become active in the prevention of CMDs, attitudes of rejection towards colleagues with CMD, and “Fair treatment of colleagues with CMD”.
- Health seeking behaviour* Two global items covering willingness to take medication and to begin a recommended psychotherapy in the case of one’s own CMD.

The questionnaire underwent a pre-test with eight employees in different professions, which on average needed 30 min for completing.

The following possible predictor variables were selected for further explorative analysis of both outcomes (see also markings in Table 1): job type, age, gender, company size, job satisfaction, mental work ability, experience within the social environment and own experience of CMDs, as well as perceived relevance of individual predisposition to develop a CMD.

**Table 1:** Sample characteristics and predictor variables for regression analysis

Item no.		Job type “Blue”, “Grey” and “White-collar workers”				Predictors included in regression models	
		Total (*n* = 610)	Blue (*n* = 193)	Grey (*n* = 169)	White (*n* = 248)	Outcome 1	Outcome 2
1	Age* (years), mean [SD]	42.0 [12.7]	45.8 [11.0]	44.5 [12.2]	37.5 [12.8]	x	x
2	Gender*^,a^ (female)	44.3 (270)	13.5 (26)	44.4 (75)	68.1 (169)	x	x
3	Origin*^,a, b^ (German-speaking countries)*	95.7 (584)	94.3 (182)	95.9 (162)	96.8 (240)		
4	Education*^,a^						
	Primary school/no education	61.1 (373)	74.6 (144)	73.4 (124)	42.3 (105)		
	Secondary school	13.9 (85)	15.5 (30)	11.8 (20)	14.1 (35)		
	High school	24.9 (152)	9.8 (19)	14.8 (25)	43.1 (108)		
5	Professional status*^,a^						
	Clerk	3.6 (22)	1.0 (2)	0.6 (1)	7.7 (19)		
	Executive activity	11.1 (68)	2.1 (4)	26.0 (44)	8.1 (29)		
	Qualified activity	22.6 (138)	3.6 (7)	8.3 (14)	47.2 (117)		
	With independent duties	14.1 (86)	9.8 (19)	5.3 (9)	23.4 (58)		
	With managerial functions	1.5 (9)	1.0 (2)	0.6 (1)	2.4 (6)		
	Worker	39.7 (242)	75.1 (145)	52.7 (89)	3.2 (8)		
	Apprenticeship	7.4 (45)	7.3 (14)	6.5 (11)	8.1 (20)		
6	Job tenure* (years), mean [SD]	10.1 [10.0]	13.8 [11.9]	7.8 [8.5]	8.8 [8.5]	x	
7	Work setting experience* (number of employers worked for)	4.3 (3.2)	4.2 (3.0)	5.4 (3.8)	3.6 (2.7)		
8	Company size*^,a^					x	x
	Very small	13.6 (83)	18.1 (35)	11.8 (20)	11.3 (28)		
	Small	26.6 (162)	28.0 (54)	33.7 (57)	20.6 (51)		
	Medium	21.1 (129)	23.8 (46)	18.3 (31)	21.0 (52)		
	Large	38.7 (236)	30.1 (58)	36.1 (61)	47.2 (117)		
9	Teamwork in the workplace^a^	80.5 (491)	80.8 (156)	73.4 (124)	85.1 (211)	x	–
10	Job satisfaction, mean [SD]^c^	2.1 [0.8]	2.1 [0.8]	2.2 [0.8]	2.1 [0.8]	x	x
11	Work ability in 2 years from now, mean [SD]^d^	2.6 [0.6]	2.6 [0.6]	2.5 [0.6]	2.7 [0.5]		
12	Mental work ability, mean [SD]^e^	2.3 [1.0]	2.2 [0.9]	2.4 [1.0]	2.3 [1.0]	x	x
13	Physical work ability, mean [SD]^e^	2.2 [1.0]	2.3 [0.9]	2.4 [1.0]	2.0 [0.9]		
14	Own experience with CMDs*^,a, f^	49.8 (304)	43.0 (83)	56.2 (95)	50.8 (126)	x	x
15	Experience with CMDs within the social environment*^,a, g^	55.1 (336)	47.7 (92)	60.9 (103)	56.9 (141)	x	x
16	Attitude of rejection towards colleagues with CMDs, mean [SD]*^,h^	1.7 [0.8]	1.8 [0.8]	1.6 [0.8]	1.7 [0.8]		
17	Willingness to be on medication in the case of own CMD, mean [SD]*^,i^	2.7 [0.8]	2.7 [0.8]	2.7 [0.9]	2.6 [0.8]		x
18	Willingness to begin recommended psychotherapy in the case of own CMD, mean [SD]*^,i^	3.2 [0.8]	3.0 [0.8]	3.2 [0.8]	3.3 [0.7]		x
19	Feeling ashamed in case of own CMD, mean [SD]*^,j^	4.3 [2.4]	4.4 [2.5]	4.2 [2.5]	4.3 [2.3]		
20	Perceived willingness of employers to become active in the prevention of CMDs, mean [SD]*^,k^	2.2 [0.8]	2.1 [0.8]	2.1 [0.8]	2.3 [0.8]		x
21	Perceived willingness of *own* employer to become active in the prevention of CMDs, mean [SD]*^,k^	2.2 [0.9]	2.1 [0.9]	2.1 [0.9]	2.4 [0.9]		
22	Fair treatment of employees with mental disorders in own company, mean [SD]*^,h^	2.7 [0.8]	2.7 [0.8]	2.6 [0.8]	2.7 [0.7]		
23	Perceived relevance of individual predisposition to develop a CMD, mean [SD]^l^	3.1 [0.6]	3.0 [0.7]	3.2 [0.6]	3.2 [0.5]	x	x
24	Perceived relevance of work-related demands (total mean score), mean [SD]^l^	3.1 [0.5]	3.0 [0.6]	3.1 [0.5]	3.1 [4.6]		x

*CMD* common mental disorder, *SD* standard deviation

*Indicates self-constructed items

^a^Percentage (cases)

^b^Germany, Switzerland, Austria

^c^Likert scaled from 1 “very satisfied” to 4 “very dissatisfied”

^d^Likert scaled from 1 “unlikely” to 3 “fairly sure”

^e^Likert scaled from 1 “very good” to 5 “very poor”

^f^“Yes” versus “no”, including “I don’t want to answer this question”: “Blue"=3.6% (n = 7), “Grey"=3.6% (n = 6), “White"=6.9% (n = 17)

^g^Categorical, “Yes”, “No” and “I don’t know”: “Blue"=18.7% (n = 36), “Grey"=15.4% (n = 26), “White"=14.5% (n = 36)

^h^Likert scaled from 1 “fully disagree” to 4 “fully agree” (“I would like to spend as little time as possible with colleagues with CMDs”)

^i^Likert scaled (1 “not at all”, 2 “probably no”, 3 “probably yes” 4 “yes, definitely”)

^j^Likert scaled from 1 “not at all” to 9 “strongly”

^k^Likert scaled from 1 “very low” to 4 “very high”

^l^Likert scaled from 1 “very irrelevant” to 4 “very relevant”

Outcome 1 “Perceived relevance of work-related CMD causes” was additionally tested for statistical influences from job tenure and teamwork in the workplace.

For outcome 2 “Perceived relevance of prevention strategies”, additionally the total mean score of outcome 1 was included in the predictor variable list, as well as the variables concerning willingness to be on medication/to begin a recommended psychotherapy in the case of one’s own CMD.

### Statistical methods

We constructed mean subscores to describe different dimensions of the outcomes 1 and 2, based on exploratory factor analysis. For both outcomes, a predominantly sufficient structure could be found with minor limitations (details are found in Online Resource 1).

The results of outcome 1 lead to the dimensions “Work content”, “Organisation of work processes” and “Interpersonal relations/leadership”, in addition to the global item “Work environment”. For outcome 2, the identified dimensions were named:

- “Work organisation”, “Coaching and training” and “Behavioural prevention” under workplace prevention, and
- “Support by specialists”, “Support by mental e-health applications” (as a synonym for e-health such as online consultation or programmes and m-health, on mobile phones, etc.) and “Support in private life” under individual prevention.

The factor analyses for the societal prevention section lead to a one-factor solution.

Score and item values will be presented descriptively (mean, standard deviation), and grouped by blue-, grey- and white-collar workers. Furthermore, descriptive indicators will be presented for each individual item.

Bivariate group differences (nominal data) were calculated using *χ*^2^ test, with the respective effect size contingency coefficient (CC). To identify relevance rankings of dimensions, differences between scores were analysed using the nonparametric Wilcoxon test. The respective effect size “*r*” was calculated by *z*/root_(cases)_ following the recommendations of Cohen (1988). Both were classified with < 0.3, < 0.5 and ≥ 0.5 indicating a low, moderate and high effect sizes (Cohen 1992).

Further analysis of the statistical influence of predictor variables was carried out for total mean scores, but not for subscores and single items. Prior to multivariate linear regression analysis with IBM SPSS 22, all selected predictors were tested on a bivariate level with *p* ≤ 0.02 and, therefore, included in the model; the variables “job type”, “age” and “gender” were adjusted in the models, regardless of their significance status. The absence of data multicollinearity was tested by variance inflation factor (VIF). Parsimonious models to minimise suppressor effects (Hosmer and Lemeshow 2000; Field 2009) will be presented, after excluding all non-significant variables by stepwise backward selection (*p*_(out)_ = 0.051). The normal distribution of residuals was proved using the Kolmogorov–Smirnov test. Model effect sizes will be discussed by means of *R*^2^ (> 0.02 low, > 0.15 moderate, > 0.35 strong (Field 2009)).

Details can be found in the study protocol in German (Burgess et al. 2017).

## Results

### Questionnaire response rate, dropouts and sample characteristics

All in all 1104 participants were contacted. 200 participants were excluded because they did not meet the inclusion criteria, and *n* = 95 due to filled quota (job type, gender, age). From the remaining 809 participants, *n* = 168 did not complete the questionnaire and *n* = 31 were excluded due to quality reasons (e.g. answering behaviour). In the end, 610 participants were included in the evaluation (75.4%): *n* = 193 blue-collar, *n* = 169 grey-collar and *n* = 248 white-collar workers. No significant differences of response rates between defined job-type groups were found. Blue-collar workers had a comparably longer job tenure (“years working for the employer”) compared to both other groups (*p* = 0.05; CC 0.29).

Study dropouts during the survey (9%) showed no job-type and item-related sample bias concerning age and gender. Considering the variables age, gender, migration background and company size, the sample is similar to the German employee population (Statistisches Bundesamt 2018). Slight distortions in some characteristics will be mentioned in the "Discussion" section. Sample characteristics can be found in Table 1.

### Respondents’ perceptions

#### Relevance of different work-related demands and individual risk factors to the development of CMDs among employees (outcome 1)

**Fig. 1:**
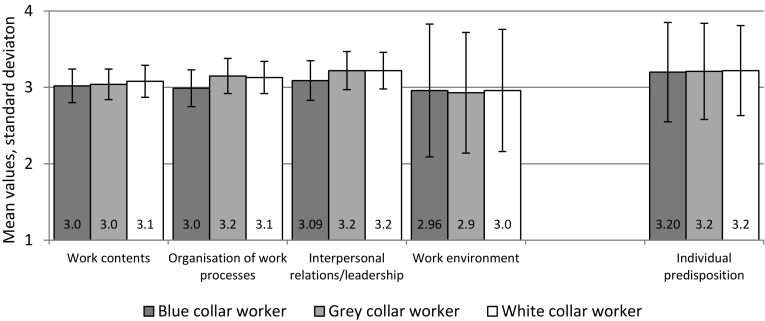
Perceived relevance of dimensions of work-related demands and individual predisposition to developing a common mental disorder (outcome 1). Mean values estimated by multivariate linear regression analysis (see Online Resource 1, Table 1) for three dimensions. “Physical work environment” and “Individual predisposition”: Single items (raw data). Possible range from 1: not relevant at all to 4: very relevant

As Fig. 1 shows, the majority of the employees surveyed—with no differences across job types—evaluated the three proposed aspects “Work content”, “Work organisation” and “Interpersonal relations/leadership” consistently as “quite” or “very relevant” to the development of CMDs. All dimension values were between 3.0 and 3.2 (SD 0.6, possible range from 1 “not relevant at all” to 4 “very relevant”).

The approval of “quite/very relevant” at item level ranged from 65% (“Organisation of work processes” among blue-collar workers) to 91% (“Lack of appreciation at work” and “Injustice at work” among grey-collar workers). Details are found in Online Resource 1, Table O1.

The global item “Physical work environment” (mean 2.9, SD 0.8) was rated a little less relevant than “Work content” and “Work organisation” (*p*_(Wilc)_ = 0.001 and 0.000, *r* = 0.13 and 0.19, respectively) and moderately less relevant than “Interpersonal relations/leadership” (*p*(Wilc) = 0.000, *r* = 0.28).

Furthermore, the latter dimension (mean 3.2, SD 0.6) was perceived slightly more relevant than “Work content” and “Work organisation” ((mean 3.1, SD 0.6, respectively): *p*_(Wilc)_ = 0.000, *r* = 0.23 and 0.17).

The relevance of an individual predisposition to CMDs was also assessed as high (global item, see Fig. 1). Blue-collar workers considered its relevance lower than white- or grey-collar workers (item approvals were 85% and 90/93%, respectively; *p*_(*χ*_^2^_)_ = 0.0023, CC 0.15). Compared with work-related demands (measured as total score, mean value 3.1, SD 0.5), the perceived relevance of individual characteristics was significantly but only slightly higher (*p*_(Wilc)_) = 0.019, *r* = 0.10).

Outcome 1 was statistically not influenced by the variables job type, age or gender in the multivariate regression model (Online Resource 2, Table O1, left side). The same could be found for the two single items “Work environment” and “Individual predisposition” concerning different job types on a bivariate level.

While the variables “Job tenure”, “Job satisfaction” and “Mental work ability” were excluded from the final regression model, people:

- working in teams,
- having personal experience with CMDs,
- having experience with CMDs within their social environment, and
- perceiving individual predisposition to developing a CMD as highly relevant

indicated a comparably higher relevance of work-related demands as a cause for developing a CMD (with the last item on the list being the strongest predictor).

The model effect size was moderate (*R*^2^ = 0.24, *df* = 9); residuals were not normally distributed (*p* = 0.000). The variance inflation factor (VIF) for assessing the absence of data multicollinearity was below 10 in all analyses.

#### Perceived relevance of prevention activities in decreasing the risk of CMDs (outcome 2)

**Fig. 2:**
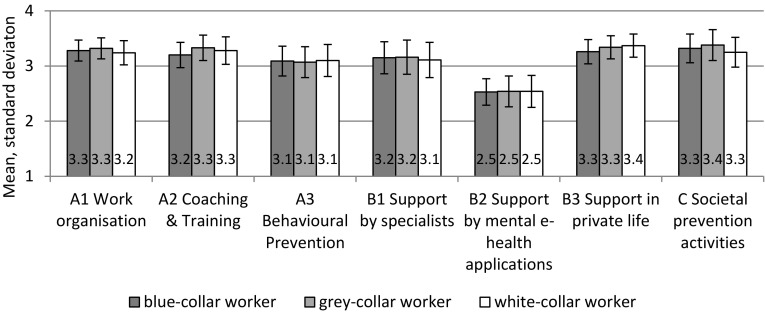
Perceived relevance of prevention activities **a** in the workplace, **b** by the individual and **c** at societal level (outcome 2). Mean score values estimated by multivariate regression analysis. Possible range from 1: very unimportant to 4: very important

All three dimensions of the topic “Workplace prevention activities” were assessed as being “relevant” or “very relevant” with mean values of at least 3.0 (Fig. 2). The approval at item level ranged from 76% for “Special initiatives by occupational health physicians” to 95% for “Planning how working time is regulated”, both among grey-collar workers.

Approvals of “Societal prevention activities” items ranged from 77% (“Public information campaigns to raise awareness of mental disorders” among white-collar workers) to 94% (“Legislation to protect employees from mental stress in the workplace” among grey-collar workers).

In the “Individual prevention” area, two out of three dimensions were rated comparably: “Support by specialists” (Seeking support from psychotherapists or general practitioner) and “Support in private life”. Item approvals were between 69% and 94% (“Expanding one’s knowledge about CMDs by reading” and “Leading a healthy lifestyle”, both among white-collar workers). “Support by mental e-health applications” (mean 2.5, SD 0.6) was rated as significantly less relevant than “Support by specialists” (mean 3.1, SD 0.5): *p*_(Wilc)_ = 0.001, *r* = 0.704 or “in private life” (mean 3.3, SD 0.5): *p* = 0.001, *r* = 0.744.

Item approvals for an online consultation with a professional and internet-based self-help programmes were 51% and 57%, respectively; perceived relevance of mobile phone apps received the lowest approval (33%). More details can be found in Online Resource 1, Tables O2–O4 for “Workplace”, “Individual” and “Societal prevention activities”.

Ranking the total score values for the three prevention areas addressed, the highest approvals were found for societal, followed by workplace and individual prevention approaches (mean 3.3/3.2/3.0), SD 0.5 each). Differences between scores were highly significant (*p*_(Wilc)_ ≤ 0.001 each) showing high effect sizes between societal (*r* = 0.66) and workplace (*r* = 0.61) compared with individual prevention.

The associated global item, where prevention should start primarily, was answered by 58.4% of the total sample with “equally in the workplace and in private life”; the remaining two fifths were fairly equally split between the two opportunities (work: 21.3%/private: 20.3%), with no differences across job types.

The results of possible predictors (adjusted for age, gender and job type) in multivariate regression models for each of the three prevention areas addressed can be summarised as follows (Table 2, outcome 2; for statistical values see Online Resource 1):

**Table 2:** Aspects influencing perceived relevance of work-related demands to the development of common mental disorders (outcome 1) and perceived relevance of prevention activities in the workplace, by individuals and at societal level (outcome 2)

Predictor variables (*)	Specification (code)	Outcome				Interpretation		
		(1) Work-related demands	(2) Prevention activities					
			(A) Workplace	(B) Individual	(C) Societal	Outcome (1)		Outcome (2)
Job type (adj.)	Blue-collar workers (1)	---	(-)	(-)	(--)	---		White-collar workers rated (A) and (C) more important than blue-collar workers
	Grey-collar workers				(--)			White-collar workers rated more important than blue- and grey-collar workers (C)
	White-collar workers (3), *RefCat*							
Age (adj.)	Years	---	(+)			---		The older the respondent, the more important (A)
Gender (adj.)	Male (1)	---				---		More important for women than men (A-C)
	Female (2)		(+)	(+)	(++)			
6 Job tenure	Years	---	<<<<	<<<<	<<<<	---		<<<<
8 Company size	Large (4)	---	(+)			---		The larger the company, the more important (A)
	Medium							
	Small							
	Very small (1), R*efCat*							
9 Teamwork in the workplace	No (1)	(+)	<<<<	<<<<	<<<<	Team workers rated outcome 1 more relevant		<<<<
	Yes (2)							
10 Job satisfaction	Very dissatisfied (4)	---				---		Respondents who are not fully satisfied rate less important than very satisfied respondents (C)
	Dissatisfied (3)							
	Satisfied (2)				(-)			
	Very satisfied (1), *RefCat*							
12 Mental work ability		---	---	---	---	---		---
14 Own experience with CMDs	No/I do not want to answer this question (1)		---	---	---	Respondents having experience rated outcome 1 more relevant		---
	Yes (2)	(+)						
15 Experience with CMDs within the social environment	No/I don’t know (1)	(+)			(+)	Respondents having experience rated outcome 1 more relevant		Respondents having experience rated (C) more important
	Yes (2)			(+)				Respondents having experience rated (B) more important
17 Willingness to be on medication in the case of own CMD	Not at all (1)	----		(-)		----		Respondents who would not be willing to be on medication in case of their own CMD rated (B) less important
	Probably no							
	Probably yes							
	Yes, definitely (4), *RefCat*							
18 Willingness to begin recommended psychotherapy in the case of own CMD	Not at all (1)	<<<<		(--)	(-)	<<<<		The less willingness to begin recommended psychotherapy, the less important (B) and (C)
	Probably no		(--)	(--)	(--)			Respondents who would not be willing to begin a recommended psychotherapy rated B and C less important
	Rather yes			(--)	(--)			
	Yes, definitely (4), *RefCat*							
20 Perceived willingness in companies to become active in the prevention of CMDs	Very low (1)	<<<<	(--)	(--)	(-)	<<<<		The less willingness in companies, the less important are prevention activities (A–C)
	Low		(--)	(--)	(-)			
	High (3)		(-)	(--)	(-)			
	Very high (4), *RefCat*							
23 Perceived relevance of individual predisposition to develop a CMD	Very significant (3)	(++)	(++)	(++)	(+)	Respondents seeing individual predisposition as significant/very significant rated outcome 1 more relevant		Respondents seeing individual predisposition as very significant rated prevention activities more important (A–C)
	Significant (2)	(++)						
	(Very) insignificant (1), *RefCat*							
24 Perceived relevance of work-related demands to CMDs (total score)		<<<<	(++)	(++)	(++)	<<<<		The more relevant, the more important were prevention activities (A-C)

Overview of multivariate linear regression results, total mean scores

*Adj.* adjusted in the regression model,* CMD* common mental disorder,* p* significance level,* RefCat* reference category (shaded grey)

Signs: (+) and (++) = positive correlation in relation to categorical code/reference category, *p* values < 0.05 and ≤ 0.001; (-) and (--) = respective negative correlation

--- = no statistical effect of predictor variable

<<<< = predictor variable not included

(*) No. referring to Table 1

- White-collar workers perceived workplace (*β* = − 0.11, *p* = 0.008) and individual prevention (*β* = − 0.13, *p* = 0.002) activities as slightly but significantly more relevant than blue-collar workers.
- Blue-collar workers (*β* = 0.15, *p* < 0.000) (as well as grey-collar workers; *β* = 0.21, *p* < 0.000) perceived societal prevention activities as more relevant than white-collar workers.
- Across all prevention areas, older respondents and women saw prevention as comparably more relevant than younger participants and men.

Furthermore, the variables “Personal experience with CMDs” and “Mental work ability” were excluded from the final model by backward elimination. The variable “Job satisfaction” showed inconsistent results: “quite satisfied” people (who were the largest group accounting for more than half of the answers) found societal prevention activities less relevant than “very satisfied” or “dissatisfied” participants.

Effects with regard to all areas of prevention were found as follows: There is a positive correlation between the relevance of prevention and:

- the perceived relevance of an individual predisposition to developing a CMD, and the willingness to begin a recommended psychotherapy in the case of one’s own CMD were declared,
- the perceived relevance of work-related demands, and
- the perceived willingness of employers to become active in the prevention of CMDs.

The following aspects influenced the answers in at least one area of prevention (see Table 2):

- The larger the company employing the respondent, the more relevant workplace prevention activities were regarded.
- Participants found societal and individual prevention activities more relevant when they had experience with CMDs within their social environment; individual efforts were evaluated as more relevant by people who were willing to be on medication.

All three model effect sizes were high (areas of prevention: A = workplace, B = individual, C = societal: *R*^2^ = 0.42/0.39/0.36, *df* = 14, respectively). Residuals were normally distributed concerning area C (relevance of societal prevention), but not concerning areas A (*p* = 0.040) and B (*p* = 0.006). The variance inflation factor (VIF) for assessing the absence of data multicollinearity was below 10 in all analyses.

## Discussion

To our knowledge, this is one of the first studies in which German employees’ perceptions about the relevance of work-related causes of common mental disorders (CMDs) and the relevance of different areas of prevention have been assessed.

A special feature of our study is the focus on German employees. In the year following our study, a population-representative survey on depression was conducted by Hegerl and Sander, but this did not only include employees. Furthermore, in contrast to our study, the evaluation was purely descriptive (Hegerl and Sander 2017).

We were also interested in the influence of working in different job types. So-called blue-, grey- and white-collar workers have different workloads and work under different conditions, which might influence relevant perceptions and attitudes. Furthermore, in a prior step we were interested in the psychometric properties and structural validity of the self-constructed items, which we summarised using specific mean scores. The main findings and implications for future research will be discussed below.

### Perceived relevance of causes for developing CMDs

The employees’ perception that “classic” workload factors such as work content, organisation of work processes and interpersonal relations (including leadership) correlate with mental health is consistent with the results described in the scientific literature (Nieuwenhuijsen et al. 2010; Harvey et al. 2017; Rothe et al. 2017). However, a causal relationship should not be derived from predominantly cross-sectional studies.

Employees seem to confirm the scientific findings with their own perspectives, and indeed across all job types. Of course, our differentiation between job types is of a gross nature, since psychomental and psychosocial workload differs between occupations, positions and general working conditions. Perceptions of workload can differ between individuals depending on their personal resources (Schaufeli and Taris 2014; Lesuffleur et al. 2014) or their health-related complaints. Several studies have shown that workers with complaints tended to attribute them to the (work) environment (i.e. Brauer et al. 2006; Magnavita 2015). Authors of other sector-differentiating workload studies found inhomogeneous results regarding the assessment of diverse perceived workloads. Divergent perception of different workloads as in leadership quality or deadline pressure (Nübling et al. 2016; Zok 2016) could not be found. At the European level, perception differences could be observed (EWCS 2017).

The relevance of an individual predisposition as the strongest of all investigated predictors might indicate that respondents think people with a higher personal vulnerability would suffer a higher strain from work-related risk factors than others. In any case, the influence of private and occupational risk factors on common mental disorders cannot be separated out due to the complex context (Weinberg and Creed 2000; Lueboonthavatchai 2009)—and employees seem to be aware of these complex interactions.

### Perceived relevance of various prevention activities to protect employees against common mental disorders

Analogously to the results of outcome 1, the majority of respondents found the suggested workplace prevention measures “very” or “quite relevant”. The same was true for activities initiated on a societal level, such as legislation to improve workplace situations, changes to the healthcare system or the provision of easily accessible counselling for people with psychological problems. Seeking support on an individual level, e.g. from psychotherapists or general practitioners or from one’s social environment, were rated a little less relevant. The lower relevance of seeking support from psychotherapists might be due to stigma reasons or due to the lack of knowledge about the effectiveness of therapy. We found a significant correlation between “Seeking support from psychotherapists” and “feeling ashamed for own CMD” in our sample (*p* ≤ 0.001, *r* = − 0.223). On the whole, employees seem to favour a multilevel approach to prevention of CMDs which is in line with official stances, e.g. the German Joint Declaration on Mental Health in the Workplace (BMAS 2013).

“Support by mental e-health applications”, namely the use of mobile phone applications, an online consultation with a professional or self-help programmes delivered over the internet, had the lowest approval level across all factors surveyed. This phenomenon is also confirmed by other investigations, e.g. Apolinário-Hagen et al. (2017a), where a lack of awareness and ambivalent or rather negative attitudes toward electronic and internet-based mental health care were found in the general population. E-mental health services are perceived as less helpful than traditional face-to-face interventions; when it comes to electronic help, therapist-assisted media health services are preferred to unguided programmes (Apolinário-Hagen et al. 2017b). We have no information about the awareness of our surveyed employees concerning e-health services. To summarise, people prefer electronic programmes which include contact with professionals, e.g. chat with a physician, compared with strictly self-use programmes. Moreover, Vollmar et al. (2017) stated in a position paper from the German Network Health Services Research that the current scientific status quo concerning health apps for treatment support suggests that risks seem to be higher than benefits. This sceptical attitude has to be taken into account when “Support by mental e-health applications” is planned as part of a comprehensive prevention approach regarding employees’ mental health.

Prevention measures on a societal level seem to be more relevant for blue- and grey-collar workers than white-collar workers showing high effect sizes between societal (*r* = 0.66) and workplace (*r* = 0.61) compared with individual prevention. This means that they attribute more effects to, or see more potential in, approaches such as legislation to protect employees from mental stress or systemic changes to the healthcare system and other institutions. Inverse trends with a lower effect size were found for prevention at workplace and individual levels. Thus, we might conclude that non-white-collar workers attribute prevention responsibilities comparably more to third parties and need more access to offers with low thresholds. We could not clarify whether this is a general phenomenon or only occurs in our study. To our knowledge, there are no other studies that confirm these findings with regard to mental health in the workplace. This is probably due to the fact that the attitudes of employees have hardly been investigated to date. All in all, the relevance of these findings should not be overestimated as the scale allows only a small variance of 1–4. Yet, it is well known that low social status is often associated with limited perceived health literacy (e.g. Berens et al. 2016). Therefore, further research is needed to gain more insight into that issue.

Furthermore, statistically significant results were found for most of the investigated predictor variables, specifically:

- *Relevance of work-related demands, age and gender* Overall, we found the strongest positive association between the perceived relevance of work-related demands to the development of CMDs and the perceived relevance of workplace prevention. Thus, employees see a need for action especially in the place that they identify as the source of problems, if also in other areas, and this view is more prevalent the older they are. Moreover, women’s generally higher perception of the relevance of prevention is in line with a generally higher health awareness than men, and a more active approach to dealing with their own CMDs, e.g. depression (Möller-Leimkühler 2002; Thompson et al. 2016). In contrast to our findings that employees attribute a high relevance to workplace interventions for preventing CMDs, recent reviews (Rothe et al. 2017; Crawford et al. 2010) have covered the limited interventional research in that field.
- *Company size* The inverse correlation with the company size predictor is seen in other studies; for example, Nübling et al. (2016) and Lai et al. (2015) found especially “good” working conditions in very small enterprises, which might influence employees’ perceptions. Furthermore, studies have shown a higher job satisfaction in smaller companies than in larger ones (García-Serrano 2011; Tansel and Gazîoğlu 2014), also based on social relationships (Azanza et al. 2013; Nübling et al. 2016).
- *Willingness for prevention in companies* It could be that a positive perception about the general willingness of employers to become active in the prevention of CMDs leads to a more optimistic assessment of prevention activities. We assume that feeling hopeful about employers’ willingness to act affects the assessment of prevention measures in a positive direction.
- *Experiences with CMDs* Previous experiences with CMDs within the social environment supported perceptions that individual and especially societal prevention activities were relevant. (The same was true for the variable “Personal experiences”, but this variable was eliminated from the final parsimonious model as a weaker correlated variable.) Of course, knowledge about and experiences with the burden of mental disorders enhances the perceived need to avoid or reduce them.
- *Help-seeking behaviour* We were able to confirm that people who indicated willingness to begin psychotherapy in the potential case of their own CMD saw particular relevance in, e.g. special initiatives by general practitioners or easily accessible counselling provision (societal prevention). Furthermore, willingness to take medication where necessary was positively correlated with the relevance of individual prevention, e.g. consulting a general practitioner. However, it should be considered that there is actually limited evidence that mental health literacy leads to help-seeking behaviour in practice (Gulliver et al. 2012; Kauer et al. 2014; Tomczyk et al. 2018).

### Strength and limitations of the study

This study is one of the first to survey the experiences and attitudes of employees with regard to CMDs in the workplace. To take into account the various burdens of different occupational groups, we decided to stratify the sample with regard to job types. As a result, more employees in our sample had “worker” positions and a lower level of education compared with the German population. The representativeness of online access panels is also limited by the typically low number of people in management positions. On the other hand, we are pleased to have data comparable with the German employee population concerning age, gender, migration background and company size. A critical discussion of strengths and limitations after surveying an online access panel can be found in Burgess et al. (2018). We favoured this approach because a significantly higher number of participants would have been necessary to generate the mandatory job-type variance and to be able to carry out the planned multivariate analyses. Also, it had to be feared that a survey performed in companies would collide with the companies’ activities related to the risk assessment of psychological stress in accordance with the German Occupational Health and Safety Act (Arbeitsschutzgesetz). In addition, there is an advantage through the possibility of non-responder analysis based on the controlling of the survey provider. The response rate was expected to be significantly higher than for postal or telephone surveys. Yet, irrespective of the way of sampling a selection bias has to be brought to mind as subjects who directly or indirectly experienced CMD might be more willing to participate in the survey than subjects with no such experience.

Our study seems to be biased in that the number of participants who had experience of mental disorders (themselves or in their social environment, confirmed by about half) was above average, compared with population-based investigations. In the latter, prevalence rates of about one quarter to one-third have been found, depending on the period under consideration (Hegerl and Sander 2017; Jacobi et al. 2014; Meyer et al. 2000). This could be attributed to the well-known fact that surveys are in general more interesting for people who are affected by the topic. It is closely related to the possibility of recall bias and may occur in studies in which participants are asked retrospectively about self-reported health problems. It might be that respondents tend to attribute psychosocial problems to the environment for no demonstrable reason. As shown in previous studies, employees attributed health problems to the work environment without traceable causes (Brauer and Mikkelsen 2010; Magnavita 2015). We checked the influence of “experience with own CMD” in our regression models. Participants with CMD rated work-related demands to be more relevant than others. The perception of people with health problems regarding workload can be different from that of healthy people. Participants affected by mental disorders could have a more negative perception of work-related factors (Ree et al. 2014). This must also be taken into account for interpretation of the importance of workplace-related risk factors. As we missed to ask about the current health status or possible sick leave at the time of the study, so we cannot draw a more precise conclusion.

We distinguished roughly between blue-, grey- and white-collar workers and their perceptions in our study. For a more detailed examination, it would be advantageous to collect more data of the working situation of individuals (e.g. working hours and shift work). In any case, our hypothesis of substantial, detectable job-type differences was not confirmed by the present survey, which should nonetheless be scrutinised. On the other hand, healthcare providers and human resource managers in our PHOEBE I-study also assessed “Interpersonal relations/leadership” as most relevant compared with work content or organisation of work processes (Junne et al. 2018).

The homogenous nature of responses from employees with different job types could be explained in the following ways:

- First, the working world in general may indeed be perceived in the same way by all employees, regardless of their experiences in their own occupation.
- Second, we cannot preclude an effect from the dimension of the question. We asked respondents to evaluate “the working world in general”, not their own company. Maybe employees reach their own limit in assessing other sectors properly, or are superficially influenced by information from the media.
- Third, it might be an artefact of respondents’ personal cost–benefit assessment in the course of a survey lasting about 30 min with a fixed incentive. The longer it takes to complete, the lower the remuneration in relation to their effort. This might lead to a superficial and undifferentiated evaluation in a sample that is mainly used to answering short market research questions.
- Finally, it could be due to a lack of knowledge and understanding about the sources and risks for the development of CMDs and respective consequences. We did not ask any questions about existing knowledge, nor did we ask for a ranking. This should be noted in further studies.

The same applies to the results on prevention. It is possible that the uniform assessments of prevention activities are due to a lack of knowledge about their impact. Here, too, we did not ask for previous knowledge.

## Conclusions

To our knowledge, the present study is the first to focus on the attitudes and opinions of employees themselves concerning CMDs at the workplace. When planning workplace prevention initiatives, activities that are accepted by employees are necessary for success. Our study shows that the awareness and acceptance of employees in this field are basically given. In Germany, many employees’ jobs are characterised by extremely low resources and/or by a high level of unfavourable strain (Rothe et al. 2017; Zok 2016). The high level of agreement on possible correlations between “bad” work and the development of a work-related CMD thus seems to correspond to reality. On the other hand, employees know exactly what “good” work should look like: after income, employment security and meaningfulness of work, social aspects of the leadership behaviour of superiors ranked fourth in the aforementioned study. In all settings, but especially in the workplace, employees see the necessity and meaningfulness of preventive measures.

For the workplace prevention of mental health problems, an Australian interdisciplinary working group has endorsed relevant and successful strategies, such as creating a positive work environment, reducing job strain, supportive change management, rewarding employees’ efforts, workplace fairness, providing support, training and mental health education, and employee responsibilities in preventing mental health problems (Reavley et al. 2014).

For this purpose, strategically oriented efforts in particular within the framework of occupational health management must be significantly intensified. This has been epidemiologically confirmed (Lüerßen et al. 2015) and was also reflected in the answers of the survey respondents. Furthermore, practical and target-group-oriented tools are lacking, as a recent review of corresponding English-language guidelines showed (Memish et al. 2017). In Germany, the “Mental Health in the Working World” project (http://psyga.info) is one initiative that has been providing comprehensive and practical tools for employers and employees for a couple of years.

Further studies which examine the existing knowledge of employees on the subject of mental disorders, especially on the effects of risk factors and on the effectiveness of preventive measures, are desirable. Therefore, a qualitative approach would be favourable due to the explorative character of these research questions. Based on the qualitative findings, the next step could be to conduct a representative survey of employees for rating certain preventive measures on their meaningfulness, to derive implications for practice.

## Electronic supplementary material

Below is the link to the electronic supplementary material.

Supplementary material 1 (PDF 481 KB)

Supplementary material 2 (PDF 503 KB)
