# Supplementary material for: Common mental disorders through the eyes of German employees: attributed relevance of work-related causes and prevention measures assessed by a standardised survey
Source: Int Arch Occup Environ Health. 2019 Feb 7;92(6):795–811. doi: 10.1007/s00420-019-01414-7 (PMC6609828; doi:10.1007/s00420-019-01414-7)
Supplement: Supplementary file 1 — Supplementary material 1 (PDF 481 KB) [file 420_2019_1414_MOESM1_ESM.pdf]

Online Resource 1: Exploratory factor analysis

Manuscript: Common mental disorders through the eyes of German employees: Attributed relevance of work-related causes and prevention measures assessed by a standardised survey- International Archives of Occupational and Environmental Health

Methods

Two outcomes were assessed concerning the structural validity and internal consistency of the investigated items:

1. Perceived "Relevance of work-related demands to the development of CMDs" and
2. Perceived "Relevance of prevention approaches at workplace, individual and societal level".

The corrected item-total correlation (CITC) as an indicator of the selectivity of the items and the overall Cronbach's α as an indicator of internal consistency were calculated for all items under outcomes 1 and 2. A Cronbach's α between 0.70 and 0.90 was considered as ideal; a CITC of 0.30 and α of 0.60 were considered the minimum for sufficient selectivity and internal consistency, respectively [Bühner 2010].

To gain information about the structural validity, all items were subjected to an exploratory factor analysis (principal component analysis (PCA), eigenvalue criterion >1, Varimax rotation). Kaiser-Meyer-Olkin criteria (KMO) test values for sampling adequacy of at least 0.5 and a significant Bartlett's sphericity test for homogeneity of variances were considered as appropriate conditions for this approach. Items were regarded as good for factor loading on their own factor if > 0.5 and on other factors if < 0.30 [de Vet et al. 2011].

Firstly, the development from self-constructed items to scores assessing the importance of relevant prevention strategies (outcome 2) was satisfied by finding good psychometric properties and structural validity.

The same was found for the dimensions of outcome 1 (perceived relevance of work-related causes to the development of CMDs) by factor analysis, but with some limitations. We accepted these in order to maintain comparability with the PHOEBE I- samples covering healthcare provider and human resource managers in further publications. In the former study, no structural validity analysis was performed.

Results

Outcome 1: Perceived relevance of different work-related demands

Sufficient factor loading on factor 1 of at least 0.50 in a 2-factor solution (explained total variance: 52.4%) was found for all items grouped under "Interpersonal relations/ leadership", although 2 out of 8 loadings just failed to match the criterion of > 0.50 on their own factor and on other factors with < 0.30 (no. 9 and 10; see Online Resource 3 (see **Table O1**)).

Most items grouped under the levels "Work content" and "Organisation of work processes" met the 0.50 criterion, but with some limitations: in total, 3 out of 6 items had higher loading than the required maximum of 0.30 on factors other than their own, and "Quantitative job demands" showed too little loading on both factors. The item "Work-privacy conflict" (level "Organisation of work processes") showed higher loading on factor 1, just reaching the 0.50 limit.

KMO and Bartlett's test values in this and in the other models had good quality. All CITC and Cronbach α values met the criteria for sufficient selectivity and internal consistency.

Online Resource 1: Exploratory factor analysis

Manuscript: Common mental disorders through the eyes of German employees: Attributed relevance of work-related causes and prevention measures assessed by a standardised survey- International Archives of Occupational and Environmental Health

Table O1: Perceived relevance of different work-related demands: Item characteristics and dimensions derived from factor analysis (outcome 1)

		Work-related demands	Total/blue-/grey-/white-collar workers		Dimensions (rotated factor component matrix) (1)	
**No**	**Dimension**	**Item**	**Mean (2)**	**SD**	**FAC1**	**FAC2**
1	Work content	Quantitative job demands	3.3/3.2/3.3/3.3	0.7/0.8/0.7/0.7	*.41*	*.47*
2		Qualitative job demands	2.9/2.9/2.9/2.9	0.8/0.8/0.8/0.8		**.71**
3		Emotional demands in the workplace	3.0/3.0/3.0/3.2	0.8/0.8/0.8/0.7	*.44*	**.52**
4		Influence and development potential on the job	2.9/2.8/2.9/2.9	0.8/0.9/0.8/0.7		**.71**
5	Organisation of work processes	Organisation of work processes	3.2/2.8/2.9/2.9	0.8/0.8/0.7/0.7		**.71**
6		Working time organisation	3.1/3.1/3.3/3.2	0.8/0.9/0.7/0.7	*.41*	**.57**
7		Work-privacy conflict	2.9/3.0/3.2/3.3	0.8/0.9/0.8/0.7	**.52**	*.40*
8	Interpersonal relations/ leadership	Communication culture in the team/in the company	3.2/3.2/3.3/3.4	0.8/0.9/0.7/0.8	**.76**	
9		Communication culture in the company	3.3/2.8/2.9/3.0	0.8/0.8/0.8/0.8	**.52**	*.36*
10		Social relationships in the workplace	2.9/2.8/3.0/2.9	0.8/0.9/0.7/0.8	**.58**	*.36*
11		Leadership quality of superiors	3.3/3.3/3.3/3.3	0.8/0.8/0.7/0.7	**.77**	
12		Leadership culture in the company	3.3/3.2/3.3/3.4	0.8/0.8/0.7/0.7	**.78**	
13		Lack of appreciation at work	3.3/3.3/3.4/3.4	0.7/0.8/0.7/0.7	**.78**	
14		Injustice at work	3.3/3.2/3.3/3.4	0.8/0.9/0.7/0.8	**.76**	
15	(Global item)	Demands of physical work environment	3.2/3.0/2.9/3.0	0.6/0.9/0.8/0.8	----	----

Legend

(1) Principal component analysis (PCA), Eigenvalue criterion > 1, Varimax rotation; loadings > 0.3 not depicted
(2) Value ranges: 4-point Likert scale 1 "no, not relevant at all" to 4 "yes, very relevant"

Abbreviations: FAC = factor; GP = general practitioner; SD = standard deviation

Statistical values: Corrected item-total correlation (CITC): "Work content" 0.46-0.51, "Organisation of work processes" 0.46-0.56, "Interpersonal relations/leadership" 0.55-0.70. Cronbach's α: "Work content" 0.71, "Organisation of work processes" 0.69, "Interpersonal relations/leadership" 0.85. KMO = 0.92, Bartlett's test p = 0.000

Explanations of items no. 1-15: Possible examples given in the questionnaire; e.g.:
1 quantitatively high demands, e.g. many tasks, take care of everything at once
2 difficult work content/technology in relation to qualification
3 dissatisfied customers
4 concerning "Influence on the job": influence on work pace, work content, work breaks; concerning "Development potential on the job": further education
5 operational processes
6 shift work, length of daily working hours
8 transparency of decisions and responsibilities
9 flat / steep hierarchy structures
10 working atmosphere in the team/in the company, mobbing
14 related to work distribution, decision-making
15 lighting conditions/noise level

Online Resource 1: Exploratory factor analysis

Manuscript: Common mental disorders through the eyes of German employees: Attributed relevance of work-related causes and prevention measures assessed by a standardised survey- International Archives of Occupational and Environmental Health

Outcome 2: Perceived relevance of workplace prevention activities

For the 17 items under "Workplace prevention activities" (see **Table O2**), a predominantly sufficient structure could be found, resulting in the three dimensions "Work organisation", "Coaching and training" and "Behavioural prevention". All factor loadings met the 0.50 loading criterion. However, some factor loadings failed to match a loading on other factors with < 0.30. All CITC values were sufficient and an ideal internal consistency was found (Cronbach α at least 0.70, with the exception of dimension B3 in Table 3, covering only two items).

Measuring "Individual prevention activities" (see **Table O3**), similar results were found, resulting in the dimensions "Support by specialists", "Mental e-health support " and "Support in private life". The only item lacking factor-loading quality ("Expanding one’s knowledge about CMDs by reading"; no. 6 in Table 3) was nonetheless included in the score construction and attributed content-wise to the dimension "Support by mental e-health applications".

For the five items under "Societal prevention activities", a 1-factor solution with sufficient psychometric properties was found (see **Table O4**).

Online Resource 1: Exploratory factor analysis

Manuscript: Common mental disorders through the eyes of German employees: Attributed relevance of work-related causes and prevention measures assessed by a standardised survey- International Archives of Occupational and Environmental Health

Table O2: Perceived relevance of workplace prevention activities: Item characteristics and dimensions derived from factor analysis (outcome 2A)

	Prevention fields	Total/blue-/grey-/white-collar workers		Dimensions (rotated factor component matrix) [1]		
**(A)**	**Workplace prevention activities**	**Mean [2]**	**SD**	**A1**	**A2**	**A3**
1	Planning how working time is regulated (e.g. breaks, shift duty, weekends, flex time)	3.4/3.3/3.6/3.2	0.7/0.7/0.6/0.6	**.727**		
2	Work environment design (e.g. noise reduction, lighting design)	3.2/3.3/3.2/3.2	0.7/0.7/0.8/0.7	**.708**		
3	New organisation of workflows ("Who does what when and in which order?")	3.2/3.2/3.2/3.2	0.7/0.7/0.7/0.6	**.703**		
4	Adjustment of requirements to the individual abilities of employees (e.g. ergonomic workstations)	3.3/3.3/3.3/3.3	0.7/0.7/0.7/0.7	**.649**		
5	Maintaining employees' leisure time (no work contact after work)	3.4/3.4/3.4/3.4	0.7/0.8/0.7/0.7	**.609**		
6	Systematic risk assessment for psychological burden (as per the German Occupational Safety and Health Act)	3.2/3.2/3.3/3.2	0.7/0.7/0.7/0.7	**.589**		*.344*
7	Improvement of the professional qualifications of employees (e.g. advanced education)	3.2/3.3/3.2/3.2	0.7/0.7/0.7/0.7	**.545**	.363	
8	Advanced education on handling common mental disorders for managers	3.4/3.4/3.5/3.4	0.7/0.8/0.6/0.7	.332	**.823**	
9	Advanced education on handling common mental disorders for human resource managers	3.4/3.4/3.5/3.4	0.7/0.8/0.7/0.7	.307	**.821**	
10	Coaching for supervisors (individual advice to strengthen and support their management tasks)	3.3/3.2/3.3/3.3	0.7/0.7/0.7/0.7		**.618**	*.430*
11	Supervision of supervisors (advice, e.g. on managing working relationships)	3.2/3.1/3.2/3.2	0.7/0.7/0.7/0.7		**.602**	*.476*
12	Advanced education on handling common mental disorders for all employees	3.2/3.1/3.2/3.2	0.7/0.8/0.7/0.8		**.580**	*.337*
13	Supervision of working teams (advice, e.g. on managing working relationships)	3.1/3.0/3.2/3.2	0.7/0.7/0.7/0.7		**.547**	*.492*
14	Offers of physical endurance training (in company or supported by company)	2.8/2.8/2.8/2.9	0.9/0.8/0.9/0.9			**.757**
15	Special initiatives from the occupational health physician (questionnaire for the early detection of mental disorders)	3.1/3.2/3.0/3.1	0.8/0.8/0.8/0.8			**.745**
16	Psychosocial counselling centre (in company or supported by company, not the occupational physician)	3.1/3.1/3.2/3.2	0.8/0.8/0.8/0.8		*.307*	**.700**
17	Stress-management programme (in company or supported by company)	3.3/3.3/3.3/3.3	0.7/0.7/0.8/0.7		*.375*	**.556**

Legend

[1] Principal component analysis (PCA), varimax rotation; eigenvalue criterion > 1 in parts A and C. In part B, a better solution was found with 3 factors in the model. Loadings > 0.3 not depicted; factors named e.g. DA1 for "Dimension 1 of prevention field A"

[2] Value ranges: 4-point Likert scale from 1 "very unimportant" to 4 "very important"

[3] Only one dimension detected.

Abbreviations: CITC = corrected item-total correlation; FAC = factor; GP = general practitioner; SD = standard deviation

Dimensions: A1 = Work organisation; A2 = Coaching & training; A3 = Behavioural prevention

Online Resource 1: Exploratory factor analysis

Manuscript: Common mental disorders through the eyes of German employees: Attributed relevance of work-related causes and prevention measures assessed by a standardised survey- International Archives of Occupational and Environmental Health

Statistical values: Explained variance: 60.5%. Corrected item-total correlation (CITC) for dimensions A1 / A2 / A3: 0.48-0.64 / 0.48-0.64 / 0.43-0.64. Cronbach's α: 0.84 / 0.87 / 0.78 (total mean score = 0.92). KMO = 0.92, Bartlett's test p = 0.000

Table O3: Perceived relevance of individual prevention activities: Item characteristics and dimensions derived from factor analysis (outcome 2B)

	**Prevention fields**	**Total/blue-/grey-/white-collar workers**		**Dimensions (rotated factor component matrix) (1)**		
**(B)**	**Individual prevention activities**	**Mean**	**SD**	**B1**	**B2**	**B3**
1	Actively seeking psychotherapy (ask GP for referral)	3.3/3.3/2.7/2.9	0.7/0.7/0.9/0.9	**.768**		
2	Consulting a psychological counselling centre in the case of problems	3.1/3.0/3.4/3.5	0.7/0.9/0.8	**.733**		
3	Consulting a general practitioner	3.3/3.4/3.4/3.4	0.7/0.8/0.7/0.6	**.729**		
4	Participation in a statutory health insurance course (e.g. stress management)	2.9/3.0/3.2/3.2	0.7/0.8/0.9/0.8	**.622**	.412	
5	Consulting an occupational health physician	3.0/3.1/3.4/3.3	0.9/0.8/0.7/0.7	**.584**		.303
6	Expanding one's knowledge about CMDs by reading (e.g. paper, journal, book)	2.8/2.8/2.6/2.6	0.7/0.7/0.8/0.8	.385	.382	.314
7	Use of mobile phone applications (support through apps in case of problems)	2.2/2.2/3.0/2.8	0.9/0.9/0.7/0.7		**.870**	
8	Use of a professional online consultation (e.g. email/chat with coach/psychotherapist)	2.5/2.5/3.0/2.8	0.8/0.8/0.7/0.7		**.824**	
9	Use of self-help programmes on the internet (independently working through)	2.6/2.6/3.2/3.1	0.8/0.8/0.7/0.8		**.779**	
10	Asking family/friends for advice and support	3.3/3.2/3.4/3.4	0.8/0.8/0.7/0.7			**.806**
11	Leading a healthy lifestyle (healthy diet, exercise, sufficient sleep)	3.4/3.3/3.3/3.3	0.7/0.7/0.8/0.8			**.741**

Legend

Information and abbreviations: see Table 2.

Dimensions: B1 = Support by specialists; B2 = Support by mental e-health applications; B3 = Support in private life

Statistical values: Explained variance: 58.8%. CITC for dimensions B1 / B2 / B3: 0.42-0.56 / 0.40-0.51 / 0.38-0.39. Cronbach's α: 0.77 / 0.76 / 0.52 (total mean score = 0.81). KMO = 0.83, Bartlett's test p = 0.000

Online Resource 1: Exploratory factor analysis

Manuscript: Common mental disorders through the eyes of German employees: Attributed relevance of work-related causes and prevention measures assessed by a standardised survey- International Archives of Occupational and Environmental Health

Table O4: Perceived relevance of societal prevention activities: Item characteristics and dimensions derived from factor analysis (outcome 2C)

	**Prevention fields**	**Total/blue-/grey-/white-collar workers**		**Dimensions (rotated factor component matrix)** [(1)]		
**(C)**	**Societal prevention activities** [(3)]	**Mean**	**SD**	**C1**		
1	Legislation to protect employees from mental stress in the workplace (e.g. in the German Occupational Safety and Health Act)	3.4/3.4/3.6/3.4	0.7/0.7/0.6/0.7	**.791**	-	-
2	Public information campaigns to raise awareness of mental disorders	3.1/3.1/3.2/3.0	0.8/0.7/0.7/0.8	**.780**	-	-
3	Change in healthcare system (e.g. short waiting time for therapy)	3.4/3.4/3.5/3.3	0.7/0.7/0.7/0.7	**.749**	-	-
4	Special initiatives from general practitioner (questionnaire for the early detection of mental problems)	3.3/3.4/3.3/3.2	0.7/0.7/0.7/0.7	**.725**	-	-
5	Easily accessible counselling for people with mental health problems (e.g. from community, church, self-help group)	3.3/3.4/3.4/3.3	0.7/0.7/0.7/0.8	**.725**	-	-

Legend

Information and abbreviations: see Table 2.

Statistical values: Explained variance: 58.8%. CITC for dimension C1: 0.56-0.64. Cronbach's α: 0.81

References

Bühner M. Introduction to the construction of test and questionnaire design [Einführung in die Test- und Fragebogenkonstruktion]. München: Pearson; 2010.

de Vet HCW, Terwee CB, Mokkink LB, Knol DL. Measurement in medicine. A practical guide. 1st ed. Cambridge: University Press; 2011.

Stephanie Burgess[1], Florian Junne [3], Eva Rothermund [2,4], Stephan Zipfel [3,] Harald Gündel [2,4], Monika A. Rieger [1], Martina Michaelis [1,5]

[1] Institute of Occupational and Social Medicine and Health Services Research, University Hospital Tübingen, Germany

[2] University Hospital for Psychosomatic Medicine and Psychotherapy, Ulm, Germany

[3] University Hospital Tübingen, Department of Psychosomatic Medicine and Psychotherapy, Germany

[4] Leadership Personality Center Ulm (LPCU), University Ulm

[5] Research Centre for Occupational and Social Medicine (FFAS), Freiburg, Germany

stephanie.burgess@med.uni-tuebingen.de
