# Supplementary material for: Common mental disorders through the eyes of German employees: attributed relevance of work-related causes and prevention measures assessed by a standardised survey
Source: Int Arch Occup Environ Health. 2019 Feb 7;92(6):795–811. doi: 10.1007/s00420-019-01414-7 (PMC6609828; doi:10.1007/s00420-019-01414-7)
Supplement: Supplementary file 2 — Supplementary material 2 (PDF 503 KB) [file 420_2019_1414_MOESM2_ESM.pdf]

Online Resource 2: Details of multivariate linear regression results

Manuscript: Common mental disorders with eyes of German employees: Attributed relevance of work-related causes and prevention measures assessed by a standardized survey– International Archives of Occupational and Environmental Health

Table O1: Aspects influencing perceived relevance of work-related demands to the development of common mental disorders (outcome 1) and perceived relevance of prevention activities at workplace, individual and societal level

(Presentation of estimated outcome mean values (total scores) and for outcome (2): variables with at least one significant result)

Variable	Specification (code)	Mean (SD)	B	Beta	p	Mean (SD)	B	Beta	p	Mean (SD)	B	Beta	p	Mean (SD)	B	Beta	p
		**(1) Perceived relevance of work-related demands to the development of CMDs [1,3]**				**(2) Perceived relevance of prevention activities in three areas [2,4]**											
						**(A) Workplace**				**(B) Individual level**				**(C) Societal level**			
Job type (adj.)	Blue-collar worker (1)	3.04 (0.60)	-0.05	-0.04	0.347	3.21 (0.52)	-0.11	**-0.11**	**0.008**	2.94 (0.47)	-0.13	**-0.13**	**0.002**	3.32 (0.57)	0.18	**0.15**	**0.000**
	Grey-collar worker	3.13 (0.46)	-0.02	-0.02	0.628	3.26 (0.46)	0.07	0.07	0.070	2.97 (0.47)	0.07	0.07	0.074	3.38 (0.52)	0.25	**0.21**	**0.000**
	White-collar worker (3), *RefCat*	3.14 (0.50)				3.22 (0.45)				2.95 (0.43)				3.25 (0.53)			
Age (adj.)		3.10 (0.51)	0.00	0.07	0.052	3.23 (0.47)	0.00	**0.07**	**0.030**	2.95 (0.45)	0.00	0.00	0.985	3.31(0.54)	0.00	0.02	0.542
Gender (adj.)	Male (1)	3.05 (0.55)	-0.01	-0.01	0.873	3.17 (0.48)	0.08	**0.09**	**0.017**	2.89 (0.46)	0.07	**0.08**	**0.041**	3.22 (0.55)	0.16	**0.15**	**0.000**
	Female (2)	3.18 (0.45)				3.30 (0.46)				3.04 (0.43)				3.42 (0.50)			
Company size	Large (4)					3.29 (0.46)	0.08	**0.08**	**0.013**								
	Medium					3.25 (0.42)											
	Small					3.17 (0.50)											
	Very small (1), *RefCat*					3.12 (0.49)											
Teamwork in the workplace	No (1)	2.99 (0.50)	0.14	**0.11**	**0.003**												
	Yes (2)	3.13 (0.50)															
Job satisfaction	Very dissatisfied (4)													3.36 (0.60)			
	Dissatisfied (3)													3.37 (0.51)			
	Satisfied (2)													3.25 (0.56)	-0.90	**-0.83**	**0.014**
	Very satisfied (1), *RefCat*													3.41 (0.47)			
Own experience with CMDs	No / I don't want to answer this question (1)	3.00 (0.55)	0.11	**0.11**	**0.005**												
	Yes (2)	3.21 (0.43)															
Experience	No / I don't know (1)	2.99 (0.55)	0.09	**0.09**	**0.028**					2.84 (0.48)				3.17 (0.53)	0.09	**0.08**	**0.022**

Online Resource 2: Details of multivariate linear regression results

Manuscript: Common mental disorders with eyes of German employees: Attributed relevance of work-related causes and prevention measures assessed by a standardized survey– International Archives of Occupational and Environmental Health

Variable	Specification (code)	Mean (SD)	B	Beta	p	Mean (SD)	B	Beta	p	Mean (SD)	B	Beta	p	Mean (SD)	B	Beta	p
		(1) Perceived relevance of work-related demands to the development of CMDs (1,3)				(2) Perceived relevance of prevention activities in three areas (2,4)											
						(A) Workplace				(B) Individual level				(C) Societal level			
with CMDs within the social environment	Yes (2)	3.20 (0.46)								3.04 (0.42)	0.07	**0.07**	**0.036**	3.42 (0.51)			
Willingness to be on medication in the case of own CMD	Not at all (1)									2.59 (0.54)	-0.17	**-0.11**	**0.005**				
	Probably no									2.92 (0.39)							
	Probably yes									2.96 (0.39)							
	Yes, definitely (4), *RefCat*									3.19 (0.51)							
Willingness to begin recommended psychotherapy in the case of own CMD	Not at all (1)					2.92 (0.58)				2.34 (0.75)	-0.41	**-0.17**	**0.000**	2.89 (0.66)	-0.32	**-0.11**	**0.002**
	Probably no					2.96 (0.48)	-0.16	**-0.11**	**0.001**	2.73 (0.42)	-0.22	**-0.15**	**0.000**	3.00 (0.54)	-0.33	**-0.20**	**0.000**
	Probably yes					3.16 (0.41)				2.90 (0.37)	-0.11	**-0.12**	**0.001**	3.22 (0.50)	-0.15	**-0.14**	**0.000**
	Yes, definitely (4), *RefCat*					3.42 (0.46)				3.15 (0.43)				3.55 (0.46)			
Perceived willingness of employers to become active in the prevention of CMDs	Very low (1)					3.20 (0.56)	-0.29	**-0.24**	**0.000**	2.85 (0.49)	-0.46	**-0.40**	**0.000**	3.32 (0.59)	-0.21	**-0.16**	**0.008**
	Low					3.20 (0.45)	-0.27	**-0.28**	**0.000**	2.92 (0.41)	-0.38	**-0.42**	**0.000**	3.27 (0.55)	-0.16	**-0.15**	**0.029**
	High					3.21 (0.44)	-0.20	**-0.18**	**0.002**	2.96 (0.45)	-0.29	**-0.27**	**0.000**	3.32 (0.48)	-0.16	**-0.13**	**0.034**
	Very high (4), *RefCat*					3.60 (0.33)				3.42 (0.41)				3.58 (0.51)			
Perceived relevance of individual predisposition to develop a CMD	Very significant (3)	3.41 (0.40)	0.73	**0.64**	**0.000**	3.52 (0.39)	0.17	**0.16**	**0.000**	3.19 (0.47)	0.13	**0.13**	**0.000**	3.48 (0.59)	0.13	**0.11**	**0.004**
	Significant (2)	3.05 (0.43)	0.39	**0.37**	**0.000**	3.16 (0.42)				2.91 (0.38)				3.23 (0.50)			
	(Very) insignificant (1), *RefCat*	2.27 (0.73)				2.79 (0.69)				2.53 (0.67)				2.96 (0.71)			
Perceived relevance of work-related demands to CMDs (total mean score)	(Score values from 1 "not relevant at all" to 4 "very relevant")					3.23 (0.47)	0.46	**0.49**	**0.000**	2.95 (0.45)	0.27	**0.31**	**0.000**	3.31 (0.54)	0.36	**0.34**	**0.000**

Online Resource 2: Details of multivariate linear regression results

Manuscript: Common mental disorders with eyes of German employees: Attributed relevance of work-related causes and prevention measures assessed by a standardized survey– International Archives of Occupational and Environmental Health

Legend

Abbreviations: adj. = adjusted in the model; B = unstandardised regression coefficient; beta standardised regression coefficient; p = significance value; SD = standard deviation; CMD = common mental disorder, df = degrees of freedom; RefCat = reference category in the case of ordinal/nominal data

(1) Predictors not significant in the final model: willingness to be on medication / begin psychotherapy in the case of own CMD, perceived willingness of employer to become active in the prevention of CMDs; company size, job satisfaction, job tenure

(2) Predictors not significant in any final model: mental work ability, own experience with CMDs

(3) R statistics: initial model (df = 19): R = 0.51, R^2 = 0.26; last model (df = 9): R = 0.49. R^2 = 0.24

(4) R statistics: (A) initial model (df = 27): R = 0.66, R^2 = 0.44; last model (df = 11): R = 0.65. R^2 = 0.42; (B) initial model (df = 27): R = 0.64, R^2 = 0.41; last model (df = 14): R = 0.63. R^2 = 0.39; (C) initial model (df = 27): R = 0.60. R^2 = 0.36; last model (df = 14): R = 0.60. R^2 = 0.36

Stephanie Burgess[1], Florian Junne [3], Eva Rothermund [2,4], Stephan Zipfel [3], Harald Gündel [2,4], Monika A. Rieger [1], Martina Michaelis [1,5]

[1] Institute of Occupational and Social Medicine and Health Services Research, University Hospital Tübingen, Germany

[2] University Hospital for Psychosomatic Medicine and Psychotherapy, Ulm, Germany

[3] University Hospital Tübingen, Department of Psychosomatic Medicine and Psychotherapy, Germany

[4] Leadership Personality Center Ulm (LPCU), University Ulm

[5] Research Centre for Occupational and Social Medicine (FFAS), Freiburg, Germany

stephanie.burgess@med.uni-tuebingen.de
